# Expanding the functional repertoire of macrophages as remote healers

**DOI:** 10.1172/JCI205823

**Published:** 2026-05-01

**Authors:** Camille Blériot, Florent Ginhoux

**Affiliations:** 1 Université Paris-Saclay, Gustave Roussy, CNRS UMR9018 Biologie du cancer et métabolisme, F-94805, Villejuif, France.; 2 Université Paris-Cité, Institut Necker Enfants Malades, CNRS UMR8253, INSERM U1151 Immunité et Métabolisme du diabète, CNRS, F-75015 Paris, France.; 3 Université Paris-Saclay, Gustave Roussy, INSERM U1356 Next Generation Immuno-Oncology Research, F-94805, Villejuif, France.; 4 Shanghai Institute of Immunology, Shanghai Jiao Tong University, Shanghai, China.; 5 Singapore Immunology Network (SIgN), Agency for Science, Technology and Research (A*STAR), Singapore.

## Abstract

The immune response is essential for maintaining host integrity, and phagocytosis is widely considered as one of its most ancient cellular functions. Accordingly, professional phagocytes such as resident tissue macrophages (RTMs) populate virtually all organs and serve as primary sentinels capable of sensing, engulfing, and eliminating invading pathogens. Yet, reflecting their early evolutionary emergence, RTMs have acquired functions that extend far beyond phagocytosis. In this issue, Salm et al. extend the macrophage toolbox, showing that macrophages residing in the peritoneal cavity function as remote healers. Using various mouse models, they demonstrated that activated peritoneal macrophages accelerate distant skin wound healing through fibronectin secretion, thereby shaping tissue repair at sites beyond their anatomical location. These findings invite us to reconsider macrophages not only as phagocytes and mediators of inflammation but also as active regulators capable of shaping extracellular architecture at a distance.

## Introduction

Wound healing is a fundamental biological process that restores tissue integrity after injury. It is typically conceptualized as a local phenomenon, occurring primarily at the site of damage and orchestrated by fibroblasts, which deposit extracellular matrix, organize provisional scaffolds, and coordinate tissue remodeling (1). While immune cells and, notably, local resident tissue macrophages (RTMs) also participate in wound healing as debris clearing agents and modulators of inflammation, the structural rebuilding of tissue has traditionally been attributed to stromal populations. This fibroblast-centric view has guided much of our understanding of tissue repair. But, in addition to the local production of healing agents by fibroblasts and myofibroblasts, plasma fibronectin derived from hepatocytes has also long been shown to contribute in the early stages of wound repair. Indeed, following vascular leakage, circulating fibronectin is incorporated into fibrin clots to form a provisional matrix that stops bleeding and prevents further tissue damage before the initiation of fibroblast-mediated repair (2). Therefore, the concept of spatial decoupling in wound healing is already recognized, but key insights in the study from Salm et al. (3) have provided evidence for the involvement of distant peritoneal macrophages, which are immune cells typically considered a priori disconnected from remote skin injuries.

## Macrophages as remote orchestrators of wound healing

Since their initial description, macrophages have been regarded as prototypical immune cells, guardians of the organism that protect the host by engulfing invaders through phagocytosis (4). However, macrophage heterogeneity in both identity and function is now well established across tissues and pathological contexts (5). Reparative RTMs in wounds, adipose tissue macrophages in metabolic diseases, or tumor-associated macrophages (TAMs) in cancer, among others, have been described and characterized, each displaying distinct, context-adapted, transcriptional and metabolic profiles. For example, TAMs favor tumor development by stimulating angiogenesis and epithelial-to-mesenchymal transitions and by maintaining an immunosuppressive environment in tumors (6). Therefore, the spectrum of RTM functions extends far beyond phagocytosis, and these cells constantly integrate signals from their immediate environment to adapt their biology. In the context of microlesions where individual cells are damaged, RTMs can extend membrane protrusions and sequester the damage, preventing initiation of an energy-demanding, potentially harmful, and ultimately futile general host immune response (7). In larger injuries, RTMs adapt their phenotype to sequentially phagocytose dead cells and any present invading pathogens, secrete factors that promote tissue repair, contribute to collagen maturation, and support the restoration of newly formed tissue (8).

**Figure 1:**
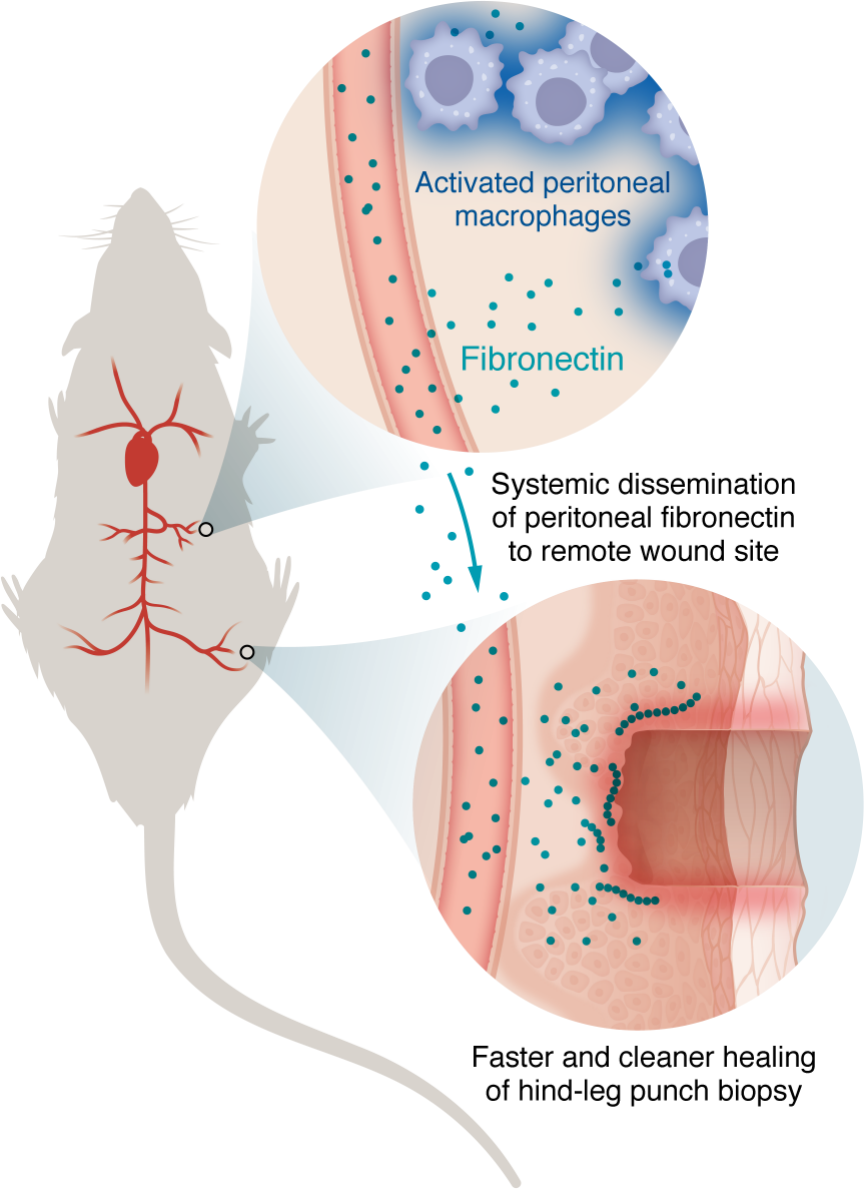
Activated peritoneal macrophages produce fibronectin to accelerate distant wound healing. In their study, Salm et al. (3) demonstrated that, upon activation by a small laparatomy, peritoneal macrophages produced fibronectin, which circulated and enhanced wound healing at a distant site.

But with this new study, Salm et al. have revealed a previously unrecognized repair function of RTMs operating at a distance (Figure 1). Briefly, they activated large peritoneal macrophages (LPMs) in a mouse model by performing a small laparotomy and studied the effects of LPM activation on a distant skin injury induced by a biopsy punch (3). Wounds in mice with activated LPMs displayed dramatically faster and cleaner resolution as compared with wounds in the nonactivated control mice. Strikingly, this effect was preserved in a parabiosis model, in which LPM activation was performed in one partner of a parabiotic pair and wound healing assessed in the other partner, supporting the notion that LPMs exert their action at a distance. While remarkable, such remote action is not entirely without precedent. Seminal studies have notably revealed that renal macrophages could regulate cardiac pressure through secretion of granulocyte-monocyte colony stimulating factor (GM-CSF) (9). Another example of distant macrophage connection is the reprograming of lung macrophages after myocardial infarction, which protects from subsequent pneumonia (10). These studies support the idea that macrophage populations do not function in isolation but rather participate in distributed interconnected organ networks.

This initial observation raised the quite natural hypothesis that LPMs migrate to the wound and act there. A prior study performed by the same group showed that LPM migration did occur in the context of sterile liver injuries (11). Indeed, in that study, LPM number declined after stimulation, consistent with the phenomenon of macrophage disappearance reaction, first described in the 1960s (12). This reaction characterizes the decline of peritoneal macrophage levels following certain inflammatory and immunological stimuli and has yet to be fully understood. In the present work, Salm et al. demonstrated that LPMs were surprisingly not recruited to the injury site, using an elegant dual recombinase/flippase LPM tracing mouse model. Of note, what is happening to activated LPMs in this context remains an intriguing open question. But a central insight from the study by Salm et al. was that macrophages promoted tissue repair without physically migrating to the site of injury. Rather, they acted through the remote secretion of fibronectin, which functions as a systemic effector of extracellular remodeling.

## Macrophage-derived fibronectin drives tissue repair

Salm et al. identified fibronectin as the soluble factor mediating LPMs action in wound healing. Combining fluorescent-tagged fibronectin and in vivo targeting of LPMs, authors provided evidence for fibronectin production by activated LPMs and its deposition within wounds. Interestingly, fibronectin secretion by LPMs may reflect a specific activation state rather than a generalized property of the lineage, and it will be important for subsequent studies to determine whether fibronectin production is linked to particular polarization states, metabolic programs, or environmental cues. For example, does IL-4–driven alternative activation enhance fibronectin transcription? Is TGF-β signaling required? Does hypoxia or mTOR signaling modulate its secretion? What is the relative importance of local and distant signals to regulate these pathways? The answers to these questions, among many others, may reveal that fibronectin production is not a generic reparative feature but a specialized adaptation under defined microenvironmental pressures.

The temporal dimension also warrants attention. During normal wound healing, fibronectin deposition is transient. As collagen accumulates and remodeling proceeds, fibronectin levels decline. If LPM-derived fibronectin persists, it could delay resolution and contribute to fibrosis in chronic wounds. This potential for fibronectin to play a maladaptive role in certain contexts highlights the need to better define the signals that trigger fibronectin production by LPMs and the regulation of that production.

Another source of questions concerns fibronectin isoforms, which were only superficially explored in the study by Salm et al. The study’s in silico analysis convincingly predicted differences between LPM-derived and liver-derived fibronectin, but further studies will need to tackle the question of which isoforms are produced, circulate, and act as effectors in wounds. Determining whether LPMs preferentially produce specific variants could clarify their biological role. Extra domain A (EDA)-containing fibronectin, for instance, can activate Toll-like receptor 4 signaling and reinforce inflammatory circuits (13) and, so, could be of particular relevance in this RTM-associated context. Thus, LPM-derived fibronectin might not simply provide structural support but also actively modulate immune responses and cell behavior.

## Clinical and translational implications

The current study by Salm et al. mostly focused on demonstrating fibronectin production by LPMs and their migration to distant wounds using various complementary mouse models. But they concluded the study with an attempt to demonstrate parallels in peritoneal fibronectin expression in patients using publicly available human peritoneal cavity single-cell transcriptomic dataset coupled with assays on patient sera. While these associative data have some limitations, which authors are the first to acknowledge, and should be considered as preliminary, they support the extension of the mouse findings to a human context and suggest fibronectin as a potential biomarker for predicting major surgical complications. It remains to be formally established what is the exact contribution of LPMs or other macrophage populations in patients, a parameter that is likely to vary depending on the underlying pathology and patient characteristics.

It is tempting to speculate about the role of macrophage-derived fibronectin in tumors, the “wounds that do not heal,” to borrow the famous assertion from H. Dvorak (14). This concept has framed decades of research with parallels between inflammation, microenvironment, and, notably, TAM activation and tumor development. Our current view is that homeostatic functions of macrophages are dysregulated in cancer, resulting in nonresolving and protumoral functions of TAMs. These latter functions have typically been understood as mediated locally by cytokines and growth factors: TGF-β, PDGF, VEGF, or IL-10, to cite a few. But Salm et al.’s findings on fibronectin production by LPMs, if confirmed in local TAMs or even in distant RTM populations, invite new perspectives. Macrophage-derived fibronectin could contribute to tumoral stroma stiffening and to the retention of cytokines and growth factors within the extracellular matrix, thereby promoting cancer cell invasion and angiogenesis. If distant macrophages can act as remote healers in patients, tumors could also exploit this capacity to generate premetastatic niches or to remodel distant stromal environments in preparation for dissemination (15). Conversely, in regenerative contexts, such as cardiac injury or diabetic foot ulcers, macrophage-derived fibronectin might accelerate repair and enhance functional recovery if properly induced. This duality reflects the broader ambivalence of wound-healing programs: regenerative when tightly regulated, yet pathological when sustained. The study by Salm et al. adds another layer of complexity by introducing distant macrophages into an already intricate equilibrium.

## Concluding remarks

Like many pioneering studies, the work by Salm et al. raises more questions than it resolves. For instance, what is the quantitative contribution of macrophage-derived fibronectin relative to fibroblast-derived fibronectin in wounds? How is fibronectin distributed spatially after secretion? How does it incorporate into existing matrices locally? What are the long-term consequences of sustained macrophage-derived fibronectin in chronic inflammatory settings? Conditional deletion of fibronectin specifically in macrophages would help clarify its impact on wound closure kinetics, angiogenesis, and scar formation. Methodologically, careful discrimination between locally synthesized and plasma-derived fibronectin will be essential. Isotope labeling, lineage tracing, and isoform-specific antibodies might be required to address these questions. Additionally, functional assays assessing cell migration and mechanical properties would strengthen the mechanistic link between fibronectin macrophage secretion and tissue remodeling outcomes. This assessment will require carefully designed experiments that overcome anticipations that omics data might not be useful in this setting (16).

In addition, the concept of remote healing may also intersect with metabolic regulation. Macrophage activation states are tightly coupled to metabolic rewiring. Glycolysis supports inflammatory programs, whereas oxidative metabolism often accompanies reparative functions. Fibronectin synthesis and secretion are energetically demanding processes. It would be informative to assess whether mitochondrial function, mTOR signaling, or nutrient availability governs fibronectin production. Such integration could reveal that matrix deposition is not merely a transcriptional event but a metabolically tuned output of immune cell state.

In conclusion, the identification of RTMs as remote healers challenges a fibroblast-centric view of wound healing and highlights the plasticity of innate immune cells. Future studies of macrophage-derived fibronectin will determine whether this phenomenon represents a specialized niche function or a generalizable principle of immune-mediated tissue organization. Whatever will emerge, the work by Salm et al. underscores an essential insight: healing is not confined to one cell type or one location but emerges from distributed networks of cellular collaboration extending beyond the boundaries we once assumed.

## Conflict of interest

The authors have declared that no conflict of interest exists.

## Funding support

The ARC Foundation (Recruiting International Leaders 2020).

- Fondation Gustave Roussy and ANR (MIAM – AAPG2023).

## Version 1 05/01/2026

Electronic publication
